# Analysis of 10 Pediatric Nephrotic Syndrome Cases With Complications of Cerebral Sinovenous Thrombosis

**DOI:** 10.3389/fped.2020.607776

**Published:** 2020-12-23

**Authors:** Liping Rong, Lizhi Chen, Zhi Dong, Hongjie Zhuang, Zhilang Lin, Ying Mo, Xiaoyun Jiang

**Affiliations:** ^1^Department of Pediatrics, The Children Kidney Center, First Affiliated Hospital, Sun Yat-sen University, Guangzhou, China; ^2^Department of Radiology, First Affiliated Hospital, Sun Yat-sen University, Guangzhou, China

**Keywords:** cerebral sinovenous thrombosis, children, nephrotic syndrome, magnetic resonance venography (MRV), complication

## Abstract

**Background:** To analyze the clinical characteristics of nephrotic syndrome (NS) with complications of cerebral sinovenous thrombosis (CSVT) in children.

**Method:** Clinical, radiographic, laboratory, and treatment data obtained from 10 confirmed cases of NS with complications of CSVT were analyzed. All patients were followed up for at least 18 months. CSVT was diagnosed by cerebral computed tomography (CT) and/or magnetic resonance imaging (MRI) with or without magnetic resonance venography (MRV) of the cerebral vessels.

**Results:** Among 10 cases reported, 4 were steroid-sensitive NS with frequent relapse, 5 were steroid-resistant (three of them had renal biopsies showing two minimal change disease and one IgA nephropathy), and 1 was steroid-sensitive with one relapse. Common clinical manifestations were headache or ophthalmodynia complicated by vomiting, dizziness, convulsion, and coma. Neuropathologic signs were positive in some cases. Papilledema appeared in only one case with winding of vein. Cerebrospinal fluid was examined in three cases with elevated pressure but normal cytological and biochemical results. D dimer and fibrinogen levels were elevated while prothrombin time and activated partial thromboplastin time were shortened. Five out of seven cases who had performed cranial CT were suspicious for cerebral thrombosis. Nine cases had cranial MRI with abnormal signs in seven cases. All of the cases received MRV, confirming the diagnosis of CVST.

**Conclusion:** Clinical manifestations of NS with CSVT are not specific but varied. Therefore, CSVT should be considered once nervous manifestations present. MRV is a better method in the diagnosis of CSVT.

## Introduction

Thrombosis is one of the common complications of nephrotic syndrome (NS). Renal veins, veins of the lower extremities, and pulmonary artery are the most common sites of thrombosis. The incidence of cerebral sinovenous thrombosis (CSVT) in children is much lower than that in adults, though the true incidence may be underestimated for many events that are asymptomatic or had a delay in diagnosis (1). However, the outcome is more serious in children. It is reported that neurologic sequelae of CSVT were apparent in up to 40% of survivors and the mortality approached 10% (2). There is few literature reporting CSVT in children with NS. Neurological symptoms of CSVT vary with an acute or subacute onset (3–10). The major manifestations of children with CSVT include convulsion and symptoms caused by intracranial hypertension such as headache, vomiting, and papilledema. However, identifying disease at early stage is difficult because these symptoms are not specific. Imaging examination is the main method to diagnose CSVT. It is suggested that confirmation of the diagnosis of CSVT relies on demonstration of thrombi in cerebral veins and/or sinuses by magnetic resonance imaging (MRI)/magnetic resonance venography (MRV) (11). Hypercoagulable state is the main cause of thromboembolism in NS. Many studies have proven that children with NS at active stage are in a hypercoagulable state (12). At present, there is no standard diagnostic criteria to identify hypercoagulable state in NS, especially there is no laboratory index for guidance on when to administer anticoagulants (13). Prophylactic anticoagulant therapy is recommended in adults NS with membranous nephropathy (11) while it remains controversial in pediatric NS regardless of the pathological types (14). Here, we reported 10 children with NS complicated by CSVT admitted to our department in recent years. The purpose of this study is to analyze the clinical characteristics of children with NS who developed CSVT.

## Materials and Methods

### Patients

Ten children with NS complicated by CSVT were included in this study. They were admitted to the children kidney disease center in the First Affiliated Hospital of Sun Yat-sen University between August 2005 and August 2020. NS was diagnosed according to the criteria defined as heavy proteinuria (urine protein > 50 mg/kg/day), hypoalbuminemia (ALB <25 g/L), hypercholesteremia (cholesterol > 5.7 mM/L), and clinical edema. We excluded patients with NS secondary to systemic disorders including systemic lupus erythematosus, hepatitis B-related nephropathy, vasculitis, and congenital NS. CSVT was diagnosed by cerebral computed tomography (CT) and/or MRI with or without MRV of the cerebral vessels (11, 15).

### Laboratory Data

Urine protein was qualitative determined in degree from negative to positive with one plus to four plus (–~ 4+). Biochemical indexes including serum albumin, cholesterin, and serum creatinine were determined by automatic biochemical analyzer. Blood coagulation function test including D dimer, prothrombin time (PT), activated partial thromboplastin time (APTT), and fibrinogen were detected with ELISA assay. Blood samples for coagulation function test were collected in negative pressure vacuum anticoagulant tube while other samples were also collected and examined in appropriate processes at the time of thrombosis.

Cases enrolled were retrospectively analyzed. Detailed clinical, radiographic, laboratory, and treatment data were obtained at the time of thrombosis. All patients have been followed up for at least 18 months.

### Ethical Statement

The study was conducted in accordance with the principles outlined in the 1964 Declaration of Helsinki and with approval from the ethics committee of the First Affiliated Hospital of Sun Yat-sen University. Written informed consent was obtained from all of the patients' parents or guardians.

## Results

### Patients' Characteristics

A total of 10 patients were enrolled in the analysis in our center over a period of 15 years (about 200 children diagnosed with NS are admitted into the center every year). There were nine males and one female aged 3 to 10 years old with an average age of 6.1 years old. There were no thrombotic events for family history or previous thrombotic events occurred in all 10 cases. Thrombotic screening with Doppler ultrasound for other sites such as lower limb veins and abdominal vessels were done in all cases and turned out to be negative. The detailed characteristics of those patients are shown in Table 1.

**Table 1:** Clinical manifestations of 10 patients with NS accompanied by CSVT.

	**Acute CSVT (≤48 h)** **(cases 1–5)**					**Subacute CSVT (48 h to 1 month)** **(cases 6–8)**			**Chronic CSVT (>1 month)** **(cases 9 and 10)**	
**Case**	**1**	**2**	**3**	**4**	**5**	**6**	**7**	**8**	**9**	**10**
Age (years)	3.0	3.3	4.9	5.0	3.0	5.9	9.0	10	9.0	7.4
Diagnosis	SSNS	SRNS(MCD)	SRNS	SRNS (IgAN)	SDNS	SRNS(MCD)	SSNS	SRNS	SSNS	SSNS
NS course	3 months	3 months	2 months	2 months	1.5 months	6 months	5 years	2 months	3.5 years	1.4 years
Drug at onset	Steroid	Steroid, Diuretic	Steroid, Diuretic	Steroid	Steroid	Steroid, Diuretic	Steroid, Diuretic	Steroid	CsA	Steroid
Edema	+	+	+	-	-	+	+	+	-	-
Hypovolemic shock/ hypovolemia	-	-	-	-	-	-	-	-	-	-
Long-term bedridden	No	No	No	No	No	No	No	No	No	No
Nervous symptomduration at onset	1 day	1 day	1 day	6 h	2 days	4 days	15 days	15 days	7 months	1 month
Symptoms	Headache, vomiting	Headache, vomiting	Ophthalmodynia, vomiting	Convulsion, coma	Headache, dizziness,	Headache, convulsion	Headache, vomiting	Headache, vomiting	Headache, dizziness, vomiting	Headache, dizziness, vomiting
Neurological signs	Stiff neck, Babinski sign (+)	None	None	Coma, no reflex	None	Coma,no reflex	None	None	Stiff neck, Babinski sign (+)	None

*SSNS, steroid-sensitive nephrotic syndrome; SRNS, steroid-resistant nephrotic syndrome; SDNS, steroid-dependent nephrotic syndrome; MCD, minimal change disease; IgAN, IgA nephropathy; CsA, Cyclosporin A*.

### Clinical Manifestations of NS

The course of NS ranged from 1.5 months to 5 years. Four cases were steroid-sensitive nephrotic syndrome (SSNS). None of the 10 patients had hypovolemic shock. For blood volume assessment, we conducted the test of passive leg raising (PLR) and rehydration test in all patients, which could predict volume responsiveness, as well as with comprehensive assessment of hematocrit, urine specific gravity, urine volume, blood pressure, and capillary refill time (CRT), none of the patients developed hypovolemia. Except for case 4, case 9, and case 10, the other seven patients had edema. Patients with SSNS were also diagnosed as frequent-relapsing NS (FRNS), among them two developed CSVT during relapses while the other two were in remission. Five cases were steroid-resistant NS (SRNS) including two cases of minimal change disease (MCD) and one case of IgA nephropathy (IgAN) confirmed by subsequent renal biopsies. There was one steroid-dependent NS (SDNS) with one relapse. At the time of CSVT being diagnosed, steroid was administered in nine cases. The other case was given Cyclosporin A (CsA) only and steroid was discontinued 1 week before neurological symptoms appeared. Four cases had received diuretic treatments. Clinical characteristics of those patients are summarized in Table 1.

### Clinical Manifestations of CSVT

CSVT could be classified into three clinical types according to the interval between onset of neurological symptoms and suspicion of CVST. Interval of <48 h, 48 h to 1 month, and more than 1 month are referred to as acute, subacute, and chronic CSVT, respectively. In this study, five patients presented with acute CSVT, in which convulsion occurred in one patient within 6 h after renal biopsy under basal anesthesia by ketamine. Three appeared as subacute and the other two patients with SSNS were chronic. CSVT was mainly manifested by headache, dizziness, convulsion, vomiting, altered consciousness, and even papilledema; these symptoms can present in isolation or in association with other symptoms. Eight out of the 10 patients presented with headache predominantly in the forehead. Headache in case nine remitted spontaneously but later recurred and progressed to paroxysmal and severe headache 1 week before admission. Case 10 had predominant pulvinar headache that occurred as paroxysm during the day but remitted when he fell asleep. Case 8 also had paroxysmal headache and had been misdiagnosed as nasosinusitis. Only one case presented with ophthalmodynia in the right eye. Seven patients had non-projectile vomiting. One patient exhibited irritability. An episode of coma had occurred in case 4 with loss of awareness and response to external stimuli. Papilledema occurred in case 9 with fundoscopy demonstrating elevation and blurring of optic disc and swelling veins along its margins without retinal hard exudates. Focal neurological deficits were not observed in patients. Meanwhile, abnormal neurological signs included low muscle tone of extremities, absent patellar tendon reflex, neck rigidity, and Babinski's sign. Yet, four patients showed no abnormal neurological signs. Nine patients had normal blood pressure except for case 5. None of them had intracranial infection (more details in Table 1).

### Laboratory Results

Blood routine, urine test, and biochemical and coagulation profiles are shown in Table 2 in detail. Heavy proteinuria was presented in the majority of patients along with severe hypoalbuminemia (<20 g/L) in six patients. Only two patients had negative urine protein at the time of CSVT. Hemoglobin levels increased markedly in five patients, in which the highest even reached 178–207 g/L in case 6. Eight patients had increased platelets counts. Renal functions were normal in all patients. In blood coagulation test, PT and APTT were shortened in five patients while fibrinogen was elevated in the other five. At the same time, levels of D dimer increased in six patients. Cerebrospinal fluid (CSF) was examined for three cases. CSF pressures were increased and reached as high as 500 mmH_2_O. However, results of cytology and biochemistry of CSF were normal.

**Table 2:** Laboratory and imaging results of 10 patients with NS accompanied by CSVT.

	**Acute CSVT (≤48 h)** **(cases 1–5)**					**Subacute CSVT (48 h to 1 month)** **(cases 6–8)**			**Chronic CSVT (>1 month)** **(cases 9 and 10)**	
**Case**	**1**	**2**	**3**	**4**	**5**	**6**	**7**	**8**	**9**	**10**
Urine protein	+ + +	+ + +	+ + + +	+ + +	+ + +	+ + +	+++	+ + + +	-	-
Urine SG	1.015	1.020	1.019	1.015	1.015	1.008	1.030	1.005	1.02	1.030
Hb (g/L)	141	167	193	135	129	133	143	159	128	164
Hct	0.407	0.451	0.553	0.401	0.350	0.371	0.426	0.431	0.347	0.421
Platelet (×10^9^/L)	583	455	247	305	494	462	95	337	326	367
Serum albumin (g/L)	21	18	15	35	19	14	12	19.6	45	34
Cholesterin (mM/L)	9.4	15.8	19.4	11.8	9.2	13.4	14.6	20.0	3.6	4.7
Serum creatinine (mM/L)	22	31	27	25	21	33	66	30	28	44
D dimer (μg/L)	1,823↑	258	1,157↑	287	233	1,451↑	3,660↑	4,000↑	4,261↑	241
PT (s)	11.6	10.6	10.2	9.9	10	12.2	13.9	10.3	13.4	11.6
APTT (s)	19↓	28.8	44.8	23.4↓	19.7↓	41.4	31.0	19.7↓	30.5	22.2↓
Fib (g/L)	8.11↑	4.35↑	6.70↑	1.64	1.32	7.44↑	4.88↑	2.36	3.98	2.62
AT III (%)	nd	nd	nd	nd	180.2	nd	nd	102.3	nd	nd
Protein C (%)	nd	nd	nd	nd	nd	nd	nd	378.8	nd	nd
Protein S (%)	nd	nd	nd	nd	nd	nd	nd	45.3	nd	nd
Vasculitis antibody	-	-	-	-	-	-	-	-	-	-
Anticardiolipin antibodies	-	-	-	-	-	-	-	-	-	-
CT	CVST	N	nd	Suspected CVST	CVST	CVST	nd	CVST	N	nd
MRI	nd	CVST	N	CVST	CVST	CVST	CVST	CVST	CVST	N
MRV	CVST	CVST	CVST	CVST	CVST	CVST	CVST	CVST	CVST	CVST
Region ofCVST	Confluence of sinuses	Straight sinus, confluence of sinuses, transverse sinus and sigmoid sinus	Superior sagittal sinus, right transverse sinus and sigmoid sinus	Extensivecerebral sinus	Superior sagittal sinus, right transverse sinus, sigmoid sinus and right jugular vein	Right transverse sinus and superior sagittal sinus	Extensive cerebral veins	Right superior sagittal sinus internal jugular vein	Extensive cerebral veins	Right superior sagittal sinus internal jugular vein

*N, normal; nd, not done; CVST, cerebral sinovenous thrombosis; Hb, hemoglobin; PT, prothrombin time; APTT, activated partial thromboplastin time; Fib, fibrinogen; AT III, antithrombin III; urine SG, urine specific gravity; HCT, hematocrit; CT, computed tomography; MRI, magnetic resonance imaging; MRV, magnetic resonance venography*.

### Imaging

**Figure 1:**
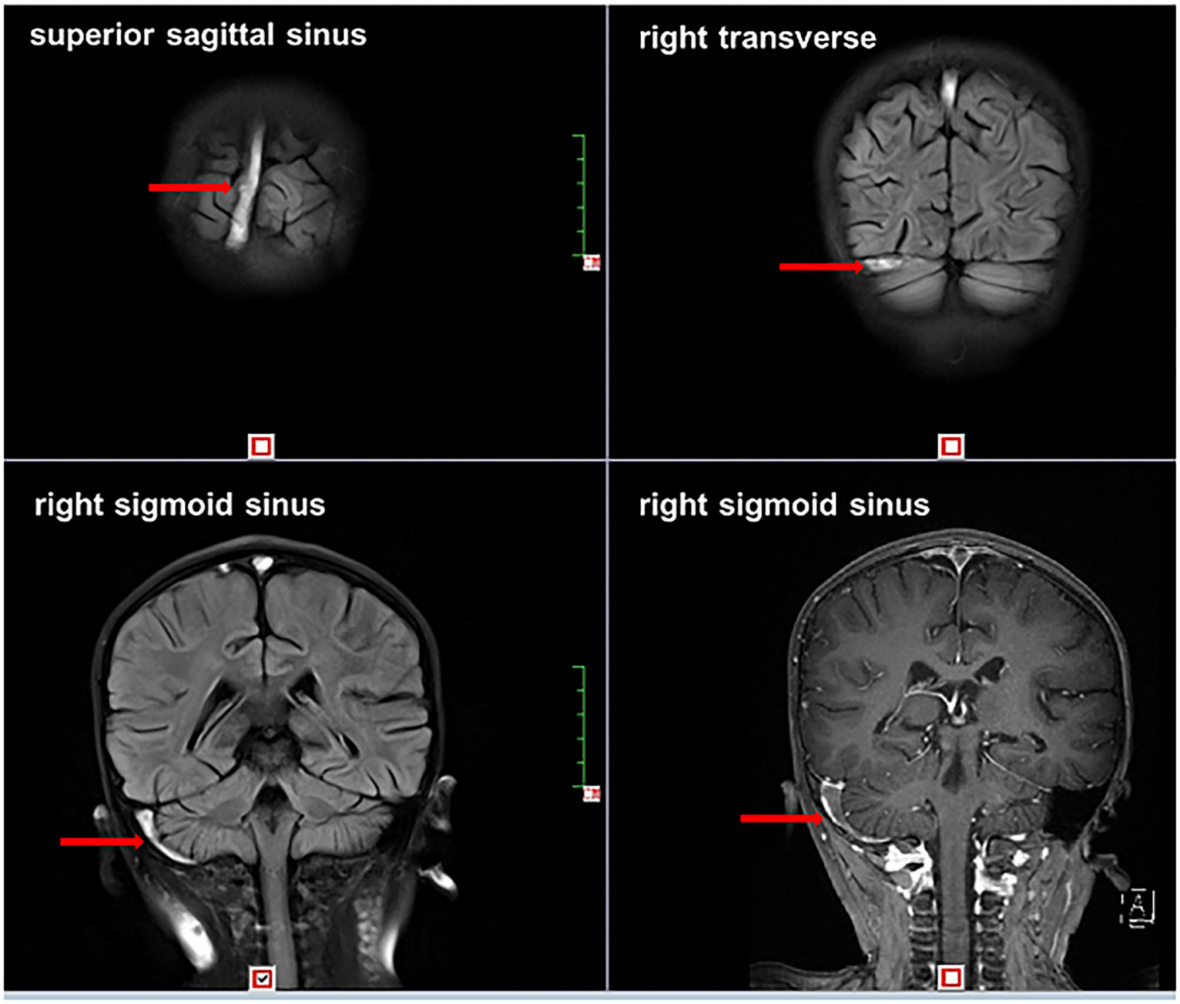
Thrombi in superior sagittal sinus, right transverse sinus, and right sigmoid sinus.

Neuroimaging of CT without contrast enhancement was performed in seven patients, two of which were absent for abnormal finding probably due to delayed scanning (≥15 days after onset of neurological symptom). Imaging of case 4 showed a low-intensity shadow in the corona radiata of posterior limb of internal capsule, which was suspected to be a flow artifact. Neuroimaging obtained by MRI was conducted in nine patients. Summary of results is listed as follows (see more details in Table 2). Case 9: gyrus was slightly swelled and sulcus was shallow. Abnormal shadow of vessels could be seen in the region of suprasellar cistern and cerebral peduncle with cavitas subarachnoidalis widened. Case 2: ventricle was slightly enlarged, sulcus and schizencephaly were slightly widened, while thrombosis was found in the left transverse sinus and sigmoid sinus. Case 6: multiple speckles were found in the right frontal lobe along with the left parietal lobe. Case 4: extensive abnormal signals distributed in the right temporal lobe, parietal lobe, and occipital lobe as well as the left parietal lobe (mainly in the right). No abnormal signals were found in case 3 and case 10. MRV was conducted in all patients; CVST was indicated in all. We compared the positive rate in diagnosing CVST among these three different image modalities conducted in our patients. The positive rates of CT, MRI, and MRA were 71.5% (5/7), 77.8% (7/9), and 100% (10/10), respectively. Thrombosis located dominantly in the superior sagittal sinus, transverse sinus, and sigmoid sinus (shown in Figure 1). Parenchymal lesions were presented in two patients described above. Digital subtraction angiography was not performed in all patients.

### Therapy

All patients were treated with intravenous low-molecular-weight heparin 62.5 U/kg every 8 h in the first 2 weeks and then reduced to every 12 h in the following 2 weeks. Oral anticoagulant warfarin was subsequently added. Dose of warfarin was adjusted to keep INR between 2.0 and 2.5. Also, dipyridamole was administered for anti-coagulation. Urokinase was used as a thrombolytic therapy as well as xueshuantong, which is a Chinese patent medicine containing extractive ingredients of herb for anti-coagulation. Meanwhile mannitol and furosemide were used to relieve intracranial hypertension. Patients with CVST symptoms were treated with low-molecular-weight dextran or albumin infusion combined with loop diuretics under the monitoring of fluid balance. One patient presented with convulsion received sedation. Specific treatments for NS were continued. Nine patients were receiving steroids, while three patients were in combination with CsA and four were in combination with tacrolimus. There was one case receiving CsA alone (more details in Table 3).

**Table 3:** Application of treatment and neurological outcome in this study.

	**Acute CSVT (≤48 h)** **(case 1–5)**					**Subacute CSVT(48 h 1 month)** **(case 6-8)**			**Chronic CSVT** **(>1 month, case 9–10)**	
**Case**	**1**	**2**	**3**	**4**	**5**	**6**	**7**	**8**	**9**	**10**
Treatment for NS	steroid +CsA	steroid +CsA	steroid +FK506	steroid	steroid +FK506	steroid +FK506	steroid	steroid +FK506	CsA	steroid +CsA
**Treatment for CVST**										
Urokinase	+	-	+	-	-	+	+	-	+	+
LMVH	+	+	+	+	+	+	+	+	+	+
Warfarin (following LMVH)	+	+	+	+	+	+	+	+	+	+
Fluid management	LMD+ diuretics	diuretics	LMD+ diuretics	diuretics	-	LMD or 20%ALB+ diuretics	LMD+ diuretics	20% ALB + diuretics	mannitol+ diuretics	mannitol + diuretics
**Time for remission of neurological symptoms**										
Headache	2 d	4 d	-	-	20 d	7 d	14 d	7 d	10 d^*^	14 d
Vomit	4 d	4 d	10 d	-	-	-	14 d	7 d	10 d	14 d
Dizziness	-	-	-	-	20 d	-	-	-	10 d	14 d
Convulsion	-	-	-	2 d	-	1 d	-	-	-	-
Coma	-	-	-	2 d	-	1 d	-	-	-	-
Ophthalmodynia	-	-	10 d	-	-	-	-	-	-	-
**Thrombus in image**										
Partial reduced	1 m	1 m	20 d	1 m	1 m	1 m	10 d	1 m	1 m	1 m
Complete reduced	6 m	6 m	3 m	>18 m^**#**^	4 m	6 m	7 m	4 m	1 y	6 m

LMVH, low molecular weight heparin; LMD, low molecular dextran [10 ml/kg.d, followed by diuretics(furosemide or torasemide)]; ALB, albumin; d, day; m, month; y, year.

* Case 9 occasionally complained of headache and dizziness.

# *Case 4 information was lacking because of withdrawal 18 months later*.

### Follow-Up

**Figure 2:**
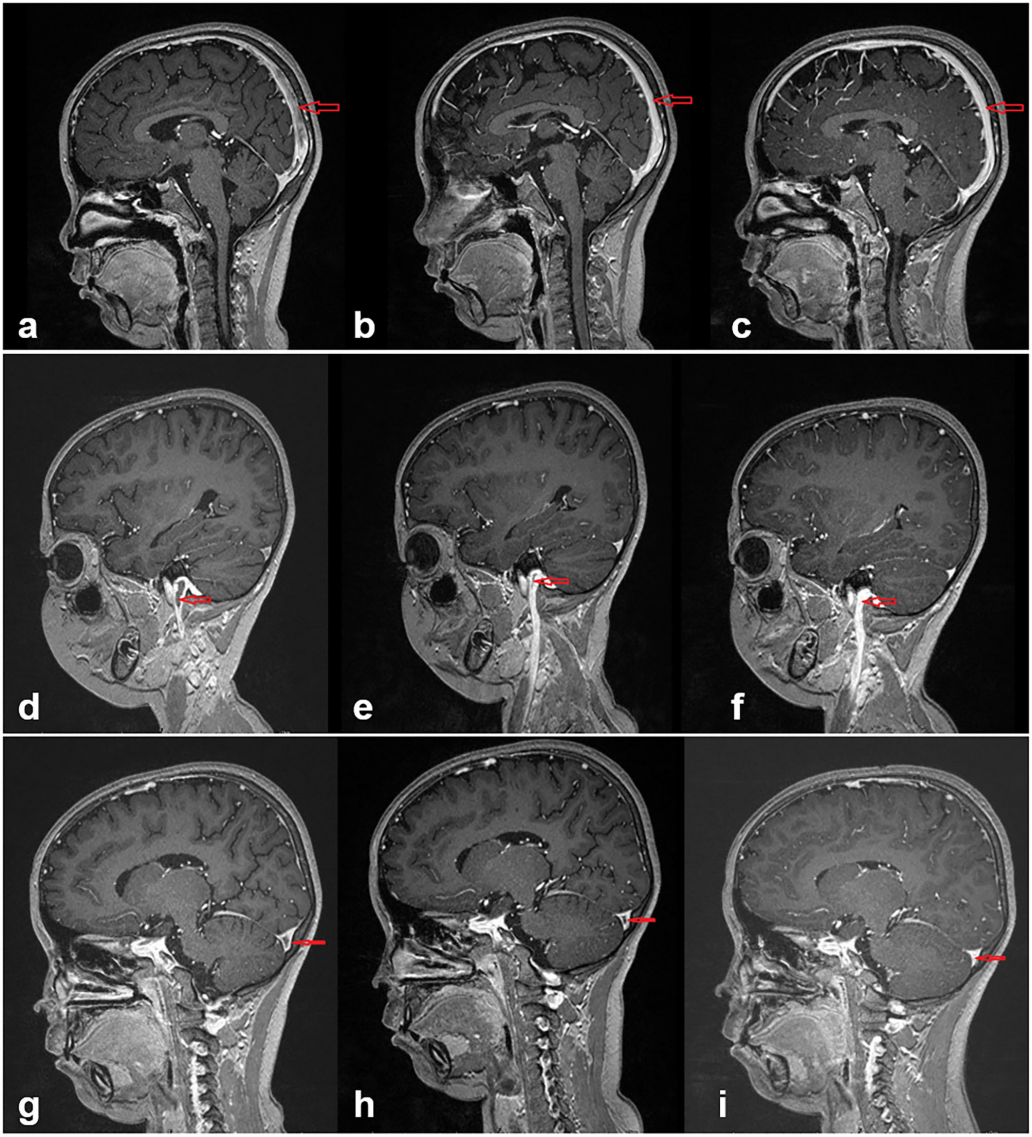
In case 8, images **(a–c)** show the changes of the sagittal sinus thrombosis at the beginning and 1 and 4 months later, respectively. With effective treatment, the thrombi had significantly reduced in the first month and disappeared 4 months later. Images **(d–f)** present in the jugular vein and images **(g–i)** present in the transverse sinus.

All patients were followed up for a mean time of 3.5 years (range, 18 months to 7.5 years). The duration of anti-coagulation therapy was 6 months. Consciousness recovered gradually from coma for Case 4, the patient was able to walk but incontinent, subsequent recovery was not available because of withdrawal 18 months later. Case 5 had fully recovered nearly 20 days after treatment. The condition of the other eight patients also improved gradually. Case 9 occasionally complained of headache and dizziness. Initial symptoms along with positive signs of nervous system all disappeared for the other cases. Urine protein was found to be negative for cases 9 and 10. For cases 1 and 2, proteinuria showed remission once CsA was administered and did not fluctuate after steroid withdrawal. For case 4, there was remission of proteinuria after 4 months. For cases 3 and 6, urine protein was persistently positive during the process of steroid reduction until tacrolimus was added. Case 7 had three relapses in 1 year; hence, tacrolimus was administered in combination with steroid, similarly in case 8. Reexamination of MRV in cases 1 and 10 showed no abnormal findings after 6 months. The thrombi became smaller in cases 5 and 7 after 1 month and 10 days, respectively. The thrombi had shrank 1 month later and disappeared 4 months later in case 8 (showed in Figure 2). Case 9 had reexamination of MRV 1 year later showing a thin left sigmoid sinus and stenosis. In the later neurological imaging studies, up to August 2020, six patients had complete recovery (more details in Table 3). There was no recurrence of thrombosis.

## Discussion

Fluss et al. reported 21 children with NS complicated by CSVT and concluded that there were seven patients with SSNS, six patients with SDNS, and four patients with SRNS. Among those cases of CSVT, 13 patients had occurred in initial onset or within 6 months of NS (6). In this study, we found that CSVT could occur in children with NS with various renal pathologies and different stages. It was not consistent with previous reports that show that thrombosis occurs mainly in children with SRNS. For adults with NS, however, thrombosis is seen more frequently in membranous nephropathy (16).

In this study, one patient had an episode of convulsion and sudden coma, three patients had headache accompanied by sudden vomiting, and two patients had recurrent headache and dizziness for several months. It can be inferred that the onset of CSVT is not only acute or subacute but also chronic in delitescence. Neurological symptoms are varied and non-specific (17). The clinical manifestations of CSVT depend on the locus of involved venous sinus, range of ischemia, the degree and speed of thrombosis, as well as the collateral circulation. Fluss et al. summarized the clinical manifestations of 21 children with NS accompanied by CSVT: 16 patients had headache, vomiting, lethargy, and irritability; convulsion was presented in 8 patients; 11 had optic disc edema; 7 had cranial nerve palsy; and 4 had hemiplegia (6). It has been reported that the main manifestations of children with CSVT are convulsion and the symptoms caused by intracranial hypertension such as headache, vomiting, and papilledema (6, 18). Headache is the main symptom in children with CSVT. Under most circumstances, headache is the initial presentation. However, confirmation of the disease at early stage is difficult because there are many factors leading to headache such as nasosinusitis, as reported in case 2, who was misdiagnosed before CVST was identified. Moreover, headache resulted from CSVT could sometimes be misdiagnosed as migraine in adults (19).

Imaging examination is the main method to diagnose CSVT. In this group, CT scan was performed in seven patients. Shadow with low intensity in the corona radiata in the posterior limb of left internal capsule was demonstrated in one patient, which was suspected to be a constructed defect. Four cases were suspected of CSVT; the other two cases were normal on CT scan. MRI was conducted for nine patients, CSVT was supported in seven patients, while the remaining were normal. CSVT was suggested in all patients who performed MRV. It is recommended that the confirmation of diagnosis of CSVT relies on the presence of thrombi in the cerebral veins and/or sinuses by MRI/MRV (11). Previous literature indicated that the diagnosis of CSVT by CT is not reliable, especially non-enhanced CT scan. Approximately 20–40% of patients with CSVT are absent for abnormal finding in CT without angiography. Even if they are found abnormal, they may be misdiagnosed as subarachnoid hemorrhage or cerebral parenchyma hemorrhage (11, 20). MRV can diagnose CSVT in an accurate and non-invasive way (11, 21). Therefore, it is recommended to perform MRV as the first-line diagnostic modality if CSVT is suspected. The results of our study support this proposal. On the other hand, although CT offers the advantages of accessibility and speed of imaging, the concern of exposure to radiation and contrast is of importance in pediatrics. MRI is often less readily available and requires more support from anesthesia/critical care to manage sedation in younger children. Therefore, sedation for lengthy MRI examination may be practically challenging, in which case CT with venogram can provide necessary data to establish the diagnosis and make rapid treatment decisions on antithrombotic therapy (14). Despite the low incidence, the diagnosis rate of CSVT in patients with NS increased in recent years in our center compared to past data. It may be partially attributed to the rapid development of neurological imaging modalities.

Hypercoagulable state is the main contributing risk factor of thromboembolism in NS (22). At present, there are no standard diagnostic criteria to identify hypercoagulable state in NS, especially since there are no laboratory indexes for guidance on when to administer anticoagulants (13). Some authors consider the hypercoagulable state as severe hypoalbuminemia (ALB <20 g/L), severe hypercholesteremia (cholesterol > 12 mM/L), and hyperfibrinogenemia (fibrinogen > 4 g/L) (23). It has been proven that severe hypoalbuminemia is the most significant biochemical risk factor (24). A previous study reported that hyperplateletosis and hyperfunction of platelet aggregability, as well as long-term use of diuretics and steroid therapy, also increase the risk of thromboembolic complications (25). In the current study, at the time CSVT was diagnosed, steroid was administered in nine cases and diuretic treatment was provided in four cases. Severe hypoalbuminemia and severe hypercholesterolemia appeared in five cases while hyperfibrinogenemia appeared in the other five cases as well as obvious increment of hemoglobin and platelet with shortening PT. Fluss et al. summarized that lack of antithrombin was the commonest abnormal coagulation index for children with NS complicated by CSVT while there was no lack of protein C or protein S (6). Due to limited laboratory testing in the past, levels of protein C and protein S were only determined in case 8, and it turned out to be without obvious abnormality. Anti-thrombin III (AT-III) was only performed in the latest two cases, yet they were within the normal reference range. Other reported risk factors involving hypovolemia and reduced activity such as being long-term bedridden did not present in our study. In the future, we will pay more attention to these laboratory tests evaluating hypercoagulation in patients with NS who are under the risk of thrombosis.

Prophylactic anticoagulant therapy is recommended to treat adult NS with membranous nephropathy (11). However, it remains controversial in the treatment of pediatric NS. Only if indexes (ALB <20 g/L, fibrinogen > 6 g/L, antithrombin <70%) are suggestive of the presence of hypercoagulable state in children with NS is conventional anticoagulant therapy advisable (23). Conversely, another group has proposed that prophylactic anticoagulant therapy should only be given to patients with thromboembolic event in the past since anticoagulants tend to cause bleeding (26). The study from Mahendranath suggested that anticoagulants merit strong consideration in pediatric CSVT (27). In our study, 4 out of 10 patients were SRNS, for whom IPNA clinical recommendations on prevention of thrombosis are made currently (28). There is insufficient evidence to recommend routine prophylactic anti-coagulation for children with SRNS and with no prior history or risk of thrombosis. Preventive anti-coagulation with low-molecular-weight heparin or oral anticoagulants is suggested in patients with a previous history of venous thromboembolic events, and treatment is considered for those with additional risk factors, including indwelling central venous lines, known hereditary thrombophilic predisposition, acute illnesses with hospitalization, infection, or risk of dehydration. Further studies need to be carried out to explore the safety and guidelines of anticoagulant therapy in pediatric NS.

The outcome appears to be good for most of the patients. It may be attributed to the early clinical suspicion followed by timely diagnosis by neurological imaging, effective anti-coagulation and thrombolytic therapy, and effective control of NS. Meanwhile, no recurrence of thrombotic event was observed during follow-up despite relapse of NS. It may be related to the adequate dosage and duration of low-molecular-weight heparin, followed by sequential therapy of warfarin, as well as regular monitoring for coagulation function.

There were some limitations in this study. The current study was a retrospective analysis and there was insufficient evaluation of coagulation status including AT-III, protein C, protein S, and examination of hereditary thrombophilic predispositions due to limited laboratory conditions. However, the significance of the tests for thrombophilia should be emphasized in children with NS associated with thrombotic events to obtain a comprehensive assessment on the etiology of their thrombosis. Therefore, more attention should be paid to the evaluation of thrombophilia in future cases. In summary, children with NS are at risk of developing thrombosis. The most common clinical manifestations of NS with CSVT are headache and vomiting, which are not specific but varied. CSVT should be considered in children with NS when neurological symptoms are presented. The positive rate in MRV was 100%, which was higher than that in CT (71.5%) and MRI (77.8%), proposing that MRV is a better modality in the diagnosis of CSVT. Symptoms and signs could be fully recovered in 3 months to a year under comprehensive therapies of anti-coagulation, thrombolysis, and control of NS. Risk factors and predictive indexes for thrombosis in NS children need to be further explored.

## Data Availability Statement

The original contributions presented in the study are included in the article/supplementary materials, further inquiries can be directed to the corresponding author/s.

## Ethics Statement

The studies involving human participants were reviewed and approved by ICE for Clinical Research and Animal Trials of the First Affiliated Hospital of Sun Yat-sen University. Written informed consent to participate in this study was provided by the participants' legal guardian/next of kin. Written informed consent was obtained from the minor(s)' legal guardian/next of kin for the publication of any potentially identifiable images or data included in this article.

## Author Contributions

YM and XJ designed the study, and reviewed and revised the manuscript. LR and LC carried out the initial analyses and drafted the initial manuscript. ZD and LR were responsible for the image collection. HZ and ZL coordinated and supervised the data collection. All authors contributed to the article and approved the submitted version.

## Conflict of Interest

The authors declare that the research was conducted in the absence of any commercial or financial relationships that could be construed as a potential conflict of interest.
